# Confirmatory factor analysis of the Mechanisms of Moral Disengagement Scale and its relationship with antisocial and delinquent behavior among justice-involved youth

**DOI:** 10.21500/20112084.8189

**Published:** 2025-12-15

**Authors:** Anyerson Stiths Gómez-Tabares, Sergio Andrés Acosta-Tobón, Daniel Camilo Aguirre-Acevedo, David Andrés Montoya-Arenas

**Affiliations:** 1 Universidad Católica Luis Amigó. Medellín, Colombia. Fundación Universitaria Luis Amigó Universidad Católica Luis Amigó Medellín Colombia; 2 Universidad de Envigado. Medellín, Colombia. Institución Universitaria de Envigado Universidad de Envigado Medellín Colombia; 3 Universidad de Antioquia. Medellín, Colombia. Universidad de Antioquia Universidad de Antioquia Medellín Colombia; 4 Universidad de San Buenaventura. Medellín, Colombia. Universidad de San Buenaventura Universidad de San Buenaventura Medellín Colombia; 5 Universidad Pontificia Bolivariana. Medellín, Colombia. Universidad Pontificia Bolivariana Universidad Pontificia Bolivariana Medellín Colombia

**Keywords:** Moral disengagement, youth, delinquency, validation, crime, Desconexión moral, juventud, delincuencia, validación, crimen

## Abstract

This study analyzed the factor solutions of the Mechanisms of Moral Disengagement Scale (MMDS) and their relationship with antisocial and delinquent behaviors in 844 justice-involved youth (*M* = 17.82 years, SD = 1.94) undergoing legal proceedings in the Colombian Juvenile Criminal Responsibility System. Participants were administered a validated Colombian version of the moral disengagement scale. Results showed that the one-, three-, four-, and eight-factor models had good goodness-of-fit indicators and acceptable internal consistency indices. However, the eight- factor structure demonstrated a superior fit to the others when the fit indices of the proposed structural models were compared. The eight-factor structure also demonstrated acceptable internal consistency and direct correlations between the factors and antisocial and criminal behaviors. These findings support that the scale is a reliable and valid measure of moral disengagement in justice-involved youth in the Colombian context.

## Resumen

Este estudio analizó las diferentes soluciones factoriales de la escala de mecanismos de desconexión moral y su relación con los comportamientos antisociales y delictivos en 844 jóvenes en conflicto con la ley (*M* = 17,82 años, DE = 1,94) que se encontraban en proceso judicial en el Sistema de Responsabilidad Penal Juvenil de Colombia. Se administró a los participantes la escala de mecanismos de desconexión moral, previamente validada en Colombia. Los resultados indicaron que los modelos de uno, tres, cuatro y ocho factores presentaron buenos indicadores de bondad de ajuste, e índices de consistencia interna aceptables. Sin embargo, al comparar los índices de ajuste de los modelos estructurales propuestos, la estructura de ocho factores demostró un ajuste superior al de los demás, una consistencia interna aceptable y correlaciones directas entre los factores y con los comportamientos antisociales y delictivos. Se puede concluir que la escala es una medida fiable y válida en términos de su estructura factorial para la medición de la desconexión moral en jóvenes en conflicto con la ley en el contexto colombiano.

## 1. Introduction

Social behavior is influenced by socio-cognitive mechanisms of moral self-regulation that aid individuals in comprehending harmful actions and their consequences for others (Bandura, 1990, 2016; Gini et al., 2014b; Gómez Tabares et al., 2024a). Moral disengagement is the process of selectively deactivating self-regulatory mechanisms to justify engagement in unethical, cruel, and inhu mane behaviors that violate self-imposed norms. This reduces personal discomfort and moral self-censorship (Bandura, 1990, 1999, 2002, 2016). Moral disengagement is the use of socio-cognitive self-justification strategies to restructure one's understanding of behavior, per sonal responsibility, consequences, and the perception of the victim as deserving harm (Gómez-Tabares et al., 2025; Moore, 2015; Lo Cricchio et al., 2022).

Moral disengagement has been extensively researched in adolescence because it is a developmental stage where significant social relationships are formed, but also a stage of psychosocial vulnerability that can lead to social risk behaviors (Durán-Palacio et al., 2023). Moral disengagement has been linked to bullying (Killer et al., 2019; Thornberg et al., 2023, 2024; Zhou et al., 2025), cyberbullying in adolescents (Orue & Calvete, 2019; Wang et al., 2016), and antisocial behavior in juvenile offenders (D'Urso et al., 2018, 2019; Gómez-Tabares et al., 2025; Petruccelli et al., 2017a, 2017b). This study examines moral disengagement among justice-involved youth because they are more likely to engage in severe antisocial behaviors and have a higher recidivism rate (Gómez-Tabares & Durán, 2024).

Research has shown that moral disengagement is strongly associated with aggressive, antisocial and delinquent behaviors in youth (D'Urso et al., 2018, 2019; Férriz Romeral et al., 2019; Petruccelli et al., 2017a; Tu et al., 2025). Additionally, moral disengagement has been found to inhibit empathy and the willingness to help victims (D'Urso et al., 2018, 2019; Eilts & Backer, 2024; Gómez-Tabares & Narváez, 2019; Petruccelli et al., 2017b). It also operates as a mediating mechanism for the effects of other psychological variables, as empathy and callous-unemotional traits (Ouvrein et al., 2018; Walters, 2018).

Meta-analyses by Gini et al. (2014b) and Férriz Romeral et al. (2019) have shown that moral disengagement predicts aggression, antisocial behavior, and delinquency, particularly in cases of serious crime. Research has also found that justice-involved youth who engage in more serious delinquent behavior, such as homicide, sexual abuse, or violent crimes, are more likely to exhibit moral disengagement. This demonstrates the relevance of moral disengagement as a construct for explaining various antisocial and delinquent behaviors in this population. Therefore, assessing moral disengagement in justice-involved youth is essential to understanding the cognitive-moral processes involved in such behavior. This highlights the importance of having valid, culturally adapted instruments for psychological research.

Moral disengagement is a determining factor in juvenile delinquency (Férriz Romeral et al., 2019). This underscores the importance of having valid, culturally adapted instruments for psychological research. However, there is a lack of studies reviewing strategies for asses- sing or measuring mechanisms of moral disengagement among justice-involved youth in Latin America. Therefore, it is important to have a reliable and valid measure with a clear factorial structure to assess moral disengagement among justice-involved youth in Colombia.

Several studies have examined the fac torial structure of the scale proposed by Bandura's theoretical model (1990, 1999, 2002, 2016). However, there is a lack of consensus regarding the dimensionality of this construct (Restrepo Cervantes et al., 2024) and a scarcity of research on this topic in the Colombian context. Therefore, it is important to conduct a study of justice-involved youth in Co lombia. Studies of adolescents use the self-report scale of moral disengagement (Bandura et al., 1996) to evaluate eight socio-cognitive mechanisms, the overall moral disengagement construct, and its relationship with affective, moral, and social variables. The scale is based on Bandura's (2016) theory of moral agency, from which the construct of moral disengagement originates.

According to the theoretical approach to moral disengagement, there are eight strategies, or socio-cognitive mechanisms, which are grouped into four moral agency "locus" (Bandura, 2016). These loci pursue specific objectives in which the mechanisms of moral disengagement operate. The behavioral locus restructures reprehen sible behavior into benevolent, "good," or acceptable behavior through the mechanisms of moral justification, euphemistic language, and advantageous com- parison. The agency locus attenuates the agentive role of the person through the mechanisms of displacement and diffusion of responsibility; the effects locus seeks to minimize, ignore, or misrepresent the consequences of harming others; and the victim locus redefines the moral condition of the victim through the mechanisms of blame attribution and dehumanization (Romera et al., 2023; Gómez-Tabares et al., 2024b; Thornberg et al., 2023).

Researchers have used this theoretical framework to validate, adapt, or design instruments that accurately reflect the nuances of moral disengagement and its influence on antisocial behavior. Stu- dies involving justice-involved youth of- ten use the self-report measure of moral disengagement (Bandura et al., 1996) to evaluate socio-cognitive mechanisms, the construct of moral disengagement, and its correlation with affective, mo ral, and social variables.

The eight-factor structure includes the following mechanisms: moral justification, euphemistic language, advantageous comparison, displacement of responsibility, diffusion of responsibility, distortion of consequences, attribution of blame, and dehumanization (Gómez-Tabares et al., 2024b; Gómez-Tabares, 2025a). Conversely, the moral agency-based structure involves an alternative model that groups the eight mechanisms of moral disengagement (Bandura, 1999, 2016). Additionally, moral disengagement has been theorized as a single socio-cognitive construct (Bandura et al., 1996; Concha Salgado et al., 2022).

In the empirical domain, the various factor structures derived from Bandura et al.'s (1996) original scale have mainly been validated in school-age adolescents with no delinquent history (see Table 1).

**Table 1:** Proposed factor structures for the moral disengagement scale

Research	Country	N	Age	Factors	Items
Bandura et al. (1996)	Italy	815	10-15	1	32
Newton et al. (2016)	Australia	452	11-15	4	22
Rubio-Garay et al. (2017)	Spain	513	15-25	3	32
García Vázquez et al. (2019)	Mexico	661	9-13	3	16
Azimpour et al. (2020)	Iran	346	22	8	32
Romera et al. (2021)	Spain	1274	11-17	4	24
Bautista et al. (2020)	Mexico	1212	11-15	8	32
Concha Salgado et al. (2022)	Chile	528	14-18	1	10
Romera et al. (2023)	Colombia	1396	11-17	8	24
	Spain	1298			
Gómez-Tabares et al. (2024)	Colombia	1434	18-30	8	32
Restrepo Cervantes et al. (2024)	Colombia	375	11-17	1	13

Psychometric studies using confirmatory factor analysis of the original scale reflect important differences in the number of fac- tors and the number of items needed to assess moral disengagement. Some studies reflect an eight-factor structure corresponding to moral disengagement mechanisms (Bautista et al., 2020; Gómez-Tabares et al., 2024b; Romera et al., 2023). Other studies have proposed structures of four factors (Bautista et al., 2020; Romera et al., 2023), three first-order factors (García Vázquez et al., 2019), and second-order grouping mechanisms (Rubio-Garay et al., 2017).

Further research has proposed statistically guided solutions to favor better indicators of model fit, which implies the elimination of items for a single-factor model (Concha Salgado et al., 2022; Restrepo Cer vantes et al., 2024). In addition, the ori ginal scale was adapted to assess similar constructs in adolescents, such as collective moral disengagement in the classroom, from a single-factor model consisting of 17 items (Gini et al., 2014a; Kollerová et al., 2018), and moral disengagement related to ethnicity, from a single-factor model consisting of 8 items (Lo Cricchio et al., 2022).

The studies that have validated the ori ginal moral disengagement scale in Latin America, and particularly in Colombia, have been with adolescents and young people immersed in educational settings and without a criminal background (Bautista et al., 2020; Concha Salgado et al., 2022; Gar cía Vásquez et al., 2019; Gómez-Tabares et al., 2024b; Restrepo Cervantes et al., 2024; Romera et al., 2023). Therefore, its psychometric properties have not been examined among justice-involved youth in Colombia.

This distinction is relevant from theoretical and empirical points of view be- cause moral disengagement mechanisms are influenced by criminal experiences, exposure to socio-legal sanctions, and sociocultural factors related to violence (Bandura, 2016; Gómez-Tabares, 2025b). Together, these factors determine the system by which youth in the justice system deactivate moral self-sanctions. Mo ral constructs are influenced by these fac- tors, so it is possible that variations in these constructs may reflect psychometric differences in relation to factor structures or the distribution of items that measure moral disengagement and its effect on antisocial and delinquent behavior in justice-involved youth in Colombia. Furthermore, the variability in the factorial structures described in the literature limits the ability to interpret evidence related to the measurement of moral disengagement and decision-making on the set of socio-cognitive mechanisms that allow justice-involved youth to distance them-selves from the moral self-sanctions of their antisocial and delinquent behaviors.

In accordance with the above, two objectives were proposed. The first was to analyze the various factorial solutions of the moral disengagement scale among Colombian justice-involved youth, based on the models proposed in the literature. This analysis is important because there is no consensus on the dimensionality of the scale (Romera et al., 2022). Furthermore, this analysis allowed us to compare the various factor solutions and deter mine the most appropriate one for assessing moral disengagement in juvenile offenders. Additionally, the internal consistency and convergent validity of the best-fitting factor structure were examined. The second objective was to analyze the associations and effects of moral disengagement on antisocial and delinquent behaviors in justice-involved youth (concurrent validity).

## 2. Method

This was a psychometric study (Ato et al., 2013) that aimed to analyze the factor structure and internal consistency of the moral disengagement scale.

### 2.1 Participants

The sample of justice-involved youth was estimated based on access to the population and the number of items that comprise the Mechanisms of Moral Disengagement Scale (MMDS). The criterion of 10 to 20 observations per estimated parameter was used to determine the sample size (Kline, 2016; Wolf et al., 2013). Due to the number of items on the scale, their ordinal nature, possible correlations between latent constructs, and the complexity of the proposed factorial models (one factor, three factors, four factors, and eight factors), a sample size of 844 justice-involved youth was determined to ensure robust parameter estimation and greater stability in confirmatory factor analyses using structural equation modeling (SEM).

Justice-involved youth were included in the study if they had been criminally sanctioned by the “Sistema de Responsabilidad Penal para Adolescentes” (SRPA) (SRPA; Juvenile Criminal Responsibility System) for crimes committed between the ages of 14 and 18. The penal sanction is imposed before the age of 18 but can be extended until the age of 25. Participants needed to have served their penal sanction in a juvenile detention facility and wanted to participate in the study voluntarily.

A total of 844 youth facing legal proceedings in Colombia's SRPA participated in the study, having been sentenced to detention for committing serious crimes or repeat offenses. The SRPA imposes sanctions on be- half of a juvenile judge, including detention in re-education centers. Three of Colombia's most important institutions were involved because they house the largest number of young people in the cities of Manizales, Medellín, Bogotá, and Montería. Of the total number of participants, 87.4% were male. The average age was 17,82 years (SD = 1.94) with an age range between 14 and 24 years. Of the total number of participants, 55.3% were in the 14 to 17 age range and 47.7% were between 18 and 24 years of age. Of the total number of juvenile offenders, 45.4% were detained in specialized centers in Medellín; 44.3%, in Bogotá; 8.1%, in Manizales; and 2.3%, in Monteria. The most common crimes were aggravated robbery (33.4%), homicide (18.1%), attempted homicide (5.7%), and drug manufacturing, traffic- king, and possession (7.3%). These crimes accounted for 65% of all offenses. Eighty-one percent of justice-involved youth reported belonging to the two lowest socioeconomic statuses (1 and 2).

### 2.2 Instruments

Mechanisms of Moral Disengagement Scale (MMDS; Bandura et al., 1996**)**. Designed to assess moral disengagement and the effect on aggressive and antisocial behavior. It consists of 32 items with a 3-point Likert scale. However, several studies have used and suggested a 5-point measure: 1 (strongly disagree) to 5 (strongly agree) (Restrepo Cervantes et al., 2024; Romera et al., 2022, 2023; Rubio-Garay et al., 2017). The scale provides a total score and eight scores for each of the moral disengagement mechanisms (moral justification, euphemistic language, advantageous comparison, displacement of responsibility, diffusion of responsibility, distortion of consequences, dehumanization, and attribution of blame).

The Scale was originally developed by Bandura et al. (1996) and has been adapted and validated in Spanish by several authors (see Table 1). There is also a validation of the scale for young people in Colombia (Gómez-Tabares et al., 2024b) with a sample of 1,431 participants (M age = 20.86, SD = 3.64). An eight-factor structure explaining 54% of the total variance was confirmed by confirmatory factor analysis (CFA). The model showed excellent fit and internal consistency, as well as metric invariance across gender and age. This study used the Spanish version validated by Gómez-Tabares et al. (2024b), which was adapted to the Colombian context. The original structure of 32 items with a 5-point Likert scale was maintained.

Antisocial and Delinquent Behavior Scale (ACBS; Andreu & Peña, 2013**)**. This scale is designed to assess the presence and severity of antisocial and delinquent behavior in young people over the past year. The scale consists of 25 items divided into five factors: pre- delinquent behavior, vandalism, property offenses, violent behavior, and alcohol and drug use. Each item is answered with a Yes or No response. Each affirmative response scores 1, for a total of 25 points. The validation study revealed a five-factor structure, with the first-order factors grouped by a second-order factor re- presenting the construct (Andreu & Peña, 2013). Moderate to high correlations were also found with reactive (r = 0.39, p < 0.05) and proactive (r = 0.61, p < 0.05) aggression (Andreu & Peña, 2013).

### 2.3 Statistical Analysis

The statistical analyses were conducted using Jamovi, an open-source software program, version 2.7 (The Jamovi project, 2025). To validate the factorial structure of the scale, structural equation modeling was performed using the SEMLj (Gallucci & Jentschke, 2021) and lavaan (Rosseel, 2019) modules. These modules are based on R packages (R Core Team, 2025) and are integrated into the Jamovi interface. These tools offer the same estimation procedures available in R, ensuring analytical consistency and transparency.

First, the sociodemographic characteristics of the justice-involved youth were described. Then, a series of confirmatory factor analyses (CFAs) were conducted to evaluate and compare alternative factorial structures of the Moral Disengagement Mechanisms Scale. The diagonally weighted least squares (DWLS) method was used for parameter estimation, which is appropriate for ordinal data. We assessed and compared models based on absolute and incremental goodness- of-fit indices (Byrne, 2016).

The fit of the models was assessed using standard goodness-of-fit indices, including the Comparative Fit Index (CFI) and the Tucker-Lewis Index (TLI). Valúes greater than .90 were considered indicative of acceptable model fit (Byrne, 2016; Hu & Bentler, 1999; McArdle & Nesselroade, 2014). In addition, root mean square error of approximation (RMSEA) and standardized root mean square residual (SRMR) were evaluated. Values below 0.08 for the RMSEA and .06 for the SRMR indicate a good model fit, suggesting minimal discrepancy between the estimated and observed covariance patterns (Hu & Bentler, 1999; McArdle & Nesselroade, 2014). Together, these indices provide a comprehensive assessment of the fit of the proposed models.

The assessment of internal consistency for the total scale and for the different factors of the proposed models of moral disengagement was conducted using McDonald's (1999) composite omega (w). The composite omega is particularly suitable for multidimensional scales because it incorporates factor loadings in its calculation (Viladrich et al., 2017). For this purpose, the component extraction method was used to identify item loadings for each factor separately in each of the proposed models (one, three, four, and eight factors). Internal consistency valúes (w) between .70 and .90 were considered acceptable, and values above .60 were also considered acceptable (Hair et al., 2021; Katz, 2006).

Convergent and concurrent validity were then analyzed. Spearman's correlation coefficient was used to examine the relationships among the scale's different factors and their correlation with anti social and delinquent behaviors. Finally, a structural equation modeling (SEM) analysis was conducted to examine the effect of moral disengagement on antiso cial and delinquent behaviors.

### 2.4 Ethical aspects

The study was approved by the Ethics Committee of the Universidad Católica Luis Amigó and the Universidad San Buenaven tura. In addition, technical approval was obtained from the Monitoring and Evaluation Subdirectorate and the Criminal Responsibility Subdirectorate of the National Directorate of the Colombian Institute of Family Welfare (Instituto Colombiano de Bienestar Familiar, ICBF) and the Psychoeducational Institute of Colombia (Instituto Psicoeducativo de Colombia, IPSICOL). The study is of minimal risk according to Resolution 8430 of 1992 of the Ministry of Social Protection. The ethical principles of research were respected, guaranteeing anonymity, voluntary participation, confidentiality, mi nimal potential for harm and transparency in the communication of results.

## 3. Results

Following Bandura's theoretical proposal (1990, 1999, 2002) and the factorial solutions reported in previous studies (see Table 1), four structural models were proposed through confirmatory factor analysis (CFA) using the DWLS method, which is appropriate in the absence of multivariate normality (Brown, 2006).

The first model corresponds to a single fac tor defining the construct of moral disengagement proposed by several authors (Bandura et al., 1996; Concha Salgado et al., 2022; Restrepo Cervantes et al., 2024). The second model is a three-factor model (Rubio-Garay et al., 2017) and the third is a four-factor model (Newton et al., 2016; Romera et al. 2021). Models 2 and 3 are theoretically guided by Bandura's (1999) proposal that the sociocognitive mechanisms of moral disengagement are oriented toward I) cognitive restructuring of behavior, II) minimizing one's agentic role, III) distorting effects or consequences, and IV) reinterpreting the role of the victim to dehumanize or blame him or her for the harm received. The three-factor model integrates the agentive role and the distortion of consequences into a single fac tor (Rubio-Garay et al., 2017).

The fourth model consists of eight factors corresponding to the eight mechanisms of moral disengagement (Azimpour et al., 2020; Bautista et al., 2020; Gómez-Tabares et al., 2024b; Romera et al., 2023). The goodness- of-fit indicators of the four proposed models of the factor structure of the moral disengagement scale are presented in Table 2.

**Table 2:** Confirmatory factor analysis fit indica- tors of the factor structure of the moral disengagement scale.

	Model 1	Model 2	Model 3	Model 4
DWLS	(1 factor)	(3 factors)	(4 factors)	(8 factors)
X^2^(df)	2416 (464) ***	2216 (461) ***	2146 (458) ***	1989 (436) ***
CFI	.973	.976	.977	.979
TLI	.971	.974	.975	.976
SRMR	.064	.061	.060	.058
RMSEA	.071	.067	.066	.065
ENT#091;CI 95%ENT#093;	ENT#091;.068- .073ENT#093;	ENT#091;.064- .070ENT#093;	ENT#091;.063- .069ENT#093;	ENT#091;.062- .068ENT#093;

*Note*. *** p < .001

The one-, three-, four-, and eight-factor structures provided acceptable fit indices in the sample of juvenile offenders. When the fit indices were compared, the four-factor model outperformed the one- and three-factor structures on the SRMR, RMSEA, CFI and TLI indices. However, the eight-factor model outperformed both the four-factor model and the one- and three-factor models in terms of optimal fit indicators (see Table 2). Therefore, the eight-factor structure was the one that provided the best fit and is consistent with the theoretical model of eight mechanisms of moral disengagement.

Table 3 displays the factor loadings and explained variance of the items for the one-, three-, and four-factor models. The items for the proposed models had a saturation greater than 0.35. When comparing models 3 and 4, it was observed that the factor loadings increased by splitting the third factor of model 2 into two different factors for model 3.

In addition, McDonald's Omega (w) was used to assess the internal consistency of the scale of the proposed models. Acceptable consistency was found for each of the factors (see Table 3 and 4).

**Table 3:** Factor loadings, internal consistency, and explained variance of the one-, three-, and four-factor models

Model 1					Model 2									Model 3			
MD			FI		FII		FIII			F1	F2		F3			F4	
Item	λ	R^2^	Item	λ	R2	λ	R2	λ	R2	λ	R2	λ	R2	λ	R2	λ	R2
MD1	.440	.194	MD1	.453	.205					.453	.205						
MD2	.635	.403	MD2	.652	.425					.652	.425						
MD3	.668	.446	MD3	.685	.469					.685	.469						
MD4	.391	.153	MD9	.536	.287					.536	.287						
MD5	.512	.262	MD10	.662	.438					.662	.438						
MD6	.646	.417	MD11	.691	.478					.691	.478						
MD7	.614	.377	MD17	.668	.446					.668	.446						
MD8	.636	.404	MD18	.688	.474					.688	.473						
MD9	.521	.272	MD19	.570	.325					.570	.325						
MD10	.644	.415	MD25	.734	.538					.734	.538						
MD11	.674	.454	MD26	.696	.484					.696	.484						
MD12	.510	.260	MD27	.701	.492					.701	.492						
MD13	.507	.257	MD4			.413	.170					.434	.188				
MD14	.420	.176	MD5			.539	.290					.564	.319				
MD15	.596	.356	MD12			.536	.287					.560	.314				
MD16	.601	.361	MD13			.535	.286					.559	.312				
MD17	.650	.422	MD20			.516	.267					.539	.291				
MD18	.669	.448	MD21			.630	.397					.658	.434				
MD19	.554	.307	MD28			.623	.388					.650	.423				
MD20	.491	.241	MD29			.584	.341					.610	.372				
MD21	.600	.359	MD6			.680	.463							.663	.440		
MD22	.560	.313	MD14			.441	.194							.431	.185		
MD23	.671	.450	MD22			.586	.344							573	.328		
MD24	.661	.437	MD30			.589	.347							.575	.330		
MD25	.715	.511	MD7 .632 .399					.632	.399							.632	.399
MD26	.678	.459	MD8 .654 .428					.654	.428							.654	.428
MD27	.683	.467	MD15 .614 .377					.614	.377							.614	.377
MD28	.592	.350	MD16 .617 .381					.617	.381							.617	.381
MD29	.555	.308	MD23 .690 .477					.690	.477							.690	.477
MD30	.560	.314	MD24 .680 .463					.680	.463							.680	.463
MD31	.563	.317	MD31 .579 .335					.579	.335							.579	.335
^ω^MD32	.635	.403	MD32 .652 .426					.652	.426							.652	.426
	.945		ω	.896		.844		.847		.896		.796		.649		.847	

*Note*. FI = disengagement by rationalization; FII = disengagement by irresponsibility; FIII = disenga- gement by depersonalization (Ru bio Garay et al., 2017). F1= cognitive restructuring of behavior; F2 =Minimizing personal agency; F3= misrepresenting or ignoring con- sequences; F4= Dehumanizing and blaming the victim (Newton et al., 2016; Romera et al., 2021).

Table 4 shows the internal consistency, factor loadings, and explained variance of the eight-factor model, which showed a better fit compared to the previous models and validated the theoretical model by reflecting the eight socio-cognitive mechanisms of moral disengagement. It also showed acceptable inter nal consistency for each factor.

**Table 4:** Factor loadings and explained variance of the eight-factor model reflecting Mechanisms of Moral Disengagement

Item	MJ		EL		AC	DR			DifR	DC			AB		DH	
	λ	R^2^	λ	R2	λ	R2	λ	R2	λ	R2	λ	R2	λ	R2	λ	R2
MD1	.482	.233														
MD9	.572	.327														
MD17	.714	.509														
MD25	.786	.617														
MD2			.650	.422												
MD10			.660	.436												
MD18			.686	.471												
MD26			.694	.482												
MD3					.651	.423										
MD11					.658	.433										
MD19					.542	.294										
MD27					.667	.444										
MD5							.553	.306								
MD13							.548	.301								
MD21							.644	.415								
MD29							.598	.357								
MD4									.418	.174						
MD12									.539	.290						
MD20									.519	.269						
MD26									.625	.390						
MD6											.663	.440				
MD14											.431	.185				
MD22											.573	.328				
MD30											.575	.330				
MD8													.651	.424		
MD16													.615	.378		
MD24													.678	.459		
MD32													.650	.422		
MD7															.678	.460
MD15															.660	.435
MD23															.744	.554
MD31															.622	.387
ω	.738		.768		.725		.677		.606		.649		.744		.772	

*Note*. MJ= moral justification; EL= euphemistic language; AC= advantageous comparison; DR= displacement of responsibility; DifR= diffusion of responsibility; DC= distortion of consequences; AB=attribution of blame; DH= dehumanization of the victim.

To provide additional evidence of convergent validity for Model 4, we examined the correlations between the eight factors corresponding to moral disengagement mechanisms (see Table 5). Moderate to large direct correlations were found between the sociocognitive mechanisms of moral disengagement (moral justification, euphemistic language, advantageous comparison, displacement of responsibility, diffusion of responsibility, distortion of consequences, attribution of blame, and dehumanization).

**Table 5:** Correlations between the eight proposed factors

	MJ	EL	AC	DR	DifR	DC	AB	DH
MJ	---							
LL	.657***	---						
AC	.641***	.714***	---					
DR	.538***	.543***	.539***	---				
DifR	474***	.495***	.497***	.624***	---			
DC	.521***	.579***	.624***	.556***	.469***	---		
AB	.550***	.646***	.667***	.536***	.511***	.589***	---	
DH	.480***	.603***	.599***	.479***	.429***	.598***	.599***	---

*p* < .001

*Note*. MJ= moral justification; EL= euphemistic language; AC= advantageous comparison; DR= displacement of responsibility; DifR= diffusion of responsibility; DC= distortion of consequences; AB=attribution of blame; DH= dehumanization of the victim.

The associations between moral disengagement mechanisms and antisocial and delinquent behaviors were analyzed. Direct and statistically significant correlations (p < .001) were found between the total score and the eight moral disengagement mechanisms with all antisocial and delinquent behaviors in justice-involved youth (see Table 6).

**Table 6:** Correlations between moral disengagement mechanisms and antisocial and delinquent behaviors.

MD	.320***	.233***	.257***	.299***	.309***	.222***
MJ	.333***	.266***	.217***	.292***	.347***	.240***
EL	.304***	.231***	.236***	.288***	.297***	.216***
AC	.253***	.192***	.196***	.243***	.254***	.157***
DifR	.219***	.151***	.177***	.202***	.189***	.172***
DR	.238***	.183***	.222***	.220***	.222***	.155***
DC	.240***	.161***	.202***	.229***	.215***	.160***
DH	.222***	.154***	.150***	.227***	.206***	.151***
AB	.181***	.118***	.151***	.171***	.177***	.139***

*p* < .001

*Note*. ADB = Antisocial and Delinquent Behavior; MJ= moral justification; EL= euphemistic Language; AC= advantageous comparison; DR= displacement of responsibility; DifR= diffusion of responsibility; DC= distortion of consequences; AB=attribution of blame; DH= dehumanization of the victim.

**Figure 1:**
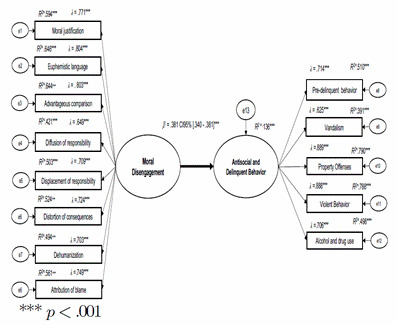
The structural effects of moral disengagement on antisocial and delinquent behavior.

Finally, a structural equation model (SEM) was proposed to analyze the effect of moral disengagement on anti social and delinquent behaviors (Figure 1). The model examined the effects of exogenous latent variables on endogenous variables. The model's goodness- of-fit indices were satisfactory (CFI = .995, TLI = .994, SRMR = .038, RM- SEA = .031 ENT#091;95% CI .021-.040ENT#093;). Moral disengagement (B = .386, SE = .0129, 3 = .360, 95% CI ENT#091;.340-.381ENT#093;, p < .001) was found to have direct effects that explained 14% of the variance in antisocial and delinquent behavior (R^2^ = .136).

This study aimed to analyze different fac torial solutions of the moral disengagement scale and their association with antisocial and delinquent behaviors among Colombian justice-involved youth. Previous literature has described factorial structures with one, three, four, or eight factors based on Bandura's (1999, 2002) proposal.

The results of this study showed acceptable fit indices for the one-, three-, four-, and eight-factor models, as well as acceptable internal consistency indices. However, when the fit indices of the proposed structural models were compared, it was observed that the eight-factor structure fit better than the others. There were moderate to large direct correlations between the factors, as well as direct and significant correlations with anti-social and delinquent behaviors.

Previous studies in young populations have compared the single-factor and multi- factor structures and have yielded conflicting results. Studies by Caprara et al. (2014), Paciello et al. (2008), and Concha Salgado et al. (2022) showed better fit for the single-factor structure compared to the eight-factor structure. Other studies have shown fit indicators supporting a three- (García Vázquez et al., 2019; Rubio-Garay et al., 2017) and four-factor solution (Newton et al., 2016; Romera et al., 2021). In contrast, the studies by Gómez-Tabares et al. (2024b) and Romera et al. (2023) demonstrated a better fit for the eight-factor structure than the single-factor

and showed that the proposed eight-factor model exhibits invariance as a function of culture, gender, and age.

Our results support various structures that are compatible with the theory (Bandura, 1990, 1999, 2002). However, the eight-factor model evidenced a better overall fit. This supports the theoretical view that moral disengagement is a multidimensional construct consisting of distinct yet interrelated sociocognitive mechanisms (Bandura, 2016). It is also consistent with previous studies that found moral disengagement to be composed of eight correlated sociocognitive mechanisms (Azimpour et al., 2020; Bautista et al., 2020; Romera et al., 2023). Each mechanism reflects a particular cognitive strategy for evading moral self-sanctions and justifying transgressive behaviors.

However, the propensity for certain mo ral disengagement mechanisms depends on the interaction of individual and socio- cultural factors related to everyday moral transgressions (Agudelo Rico et al., 2024; Caprara et al., 2009; Gómez Tabares, 2025b). This could explain discrepancies in factor structures across studies with culturally diverse samples.

Groups that exhibit less heterogeneity in antisocial behavior, such as children, adolescents, and young people with no criminal history, tend to commit less frequent, less serious, and more socially controlled moral transgressions compared to justice-involved youth. This could reduce the dispersion of responses to the Moral Disengagement (MMD) scale items corresponding to each of the eight mechanisms and, therefore, the possibility of differentiating between them empirically.

Previous studies have reported these differences when comparing moral disengagement mechanism scores between justice-involved youth (juvenile offenders or formerly recruited youth) and school-attending adolescents with no criminal record (Agudelo Rico et al., 2024; Gómez-Tabares & Durán, 2021). The justice-involved youth groups had statistically higher average scores, with medium to large effect sizes, than the school-attending group on moral disengagement mechanisms. These differences are largely attributed to variability in previous experiences in each group, particularly in terms of early exposure to violence and social legitimization of aggression, which tends to result in greater deactivation of moral norms (Bandura, 2016; Gómez-Tabares & Durán, 2021).

Our study found positive and significant associations between the eight mechanisms of moral disengagement and various antisocial and delinquent behaviors. The proposed structural equation model (SEM) showed that mo ral disengagement explains antisocial and delinquent behaviors in justice-involved youth. These findings support the idea that moral disengagement is a stable predictor of antisocial behaviors in justice-involved youth (D'Urso et al., 2018; Kiriakidis, 2008; Paciello et al., 2017; Petruccelli et al., 2017a, 2017b; Shulman et al., 2011) and that its effect is greater in cases of serious violent crimes (Férriz et al., 2019).

Both loci and mechanisms of moral disengagement perform unique functions of exoneration from moral transgressions (Caprara et al., 2009), so previous antisocial and delinquent experiences increase interindividual variability in psychometrically detecting the multidimensionality of cognitive processes of moral disengagement. In this sense, justice-involved youth are more exposed to social violence and have been involved in serious crimes or are repeat offenders (Férriz Romeral et al., 2019; Gómez Tabares, 2025b), increasing the statistical possibility of discriminating among the various sociocognitive structures in the set of items on the scale to deactivate the moral regulation system in the face of heterogeneity in antisocial and delinquent behaviors.

The variability in the structure of moral disengagement may also be influenced by cultural factors. For example, Romera et al. (2023) found differences in the facto rial structure of the moral disengagement scale among Colombian and Spanish adolescents. The overall fit indices were acceptable for the one-, four-, and eight-factor models in the Spanish sample. However, in the Colombian sample, only the eight-factor model showed a good fit. Chi-square difference tests supported an eight-factor structure in both samples. The authors argue that these differences in the scale's factorial structure are due to cultural fac- tors related to each society's disciplinary control to regulate transgressive behavior. These differences could explain the lack of consensus on the factorial structure of the scale in Italy (Bandura et al., 1996; Caprara et al., 2014), Spain (Romera et al., 2021; Rubio-Garay et al., 2017), and Latin America (Bautista et al., 2020; Gómez-Tabares et al., 2024; Romera et al., 2023), and highlight the need to analyze moral disengagement from an intercultural perspective.

Conversely, evidence that moral disengagement is a multifactorial construct enables us to study its eight specific mechanisms in justice-involved youth in Colombia and analyze how it interrelates with different transgressive behaviors. The results indicate that the scale is a reliable self-report measure of moral disengagement mechanisms among justice-involved youth in Colombia. Although previous studies have employed the instrument to examine moral disengagement in this population (Cabrera et al., 2020; Gómez-Tabares & Durán, 2021, 2024b; Gómez-Tabares & Narváez, 2019), this is the first study to test its factorial structure and provide empirical evidence supporting its validity for assessing moral disengagement mechanisms within this context.

The CFA results, internal consistency, and correlations between its factors and antisocial and delinquent behaviors provide evidence that moral disengagement is a socio-cognitive process that uses different independent strategies to justify immoral, transgressive, and inhumane behaviors. Thus, moral disengagement is a multifactorial construct defined by these strategies. The tendency toward moral disengagement is facilitated by various socio-cognitive mechanisms that function as resetting strategies of the moral regulatory system and enable transgressive behaviors (Moore, 2015, Bandura, 2016).

From a theoretical perspective, three- and four-factor structures are useful to reflect the domains that group the goals of moral disengagement, which include restructuring immoral behavior, minimizing the agentic role, distorting consequences, and blaming the victim (Bandura, 1999, 2002). However, an eight-factor structure more accurately reflects the tendency to use specific sociocognitive mechanisms in different situations to selectively disengage the moral self-regulatory system (Bandura, 2016).

Studies suggesting that moral disengagement is a unidimensional construct (Caprara et al., 2014; Concha Salgado et al., 2022; Lo Cricchio et al., 2022) support the idea that moral disengagement is a unique and relatively stable trait that predisposes people to justify transgressive behavior. Our findings do not allow us to completely rule out the possibility that moral disengagement is a unidimensional construct. However, a multidimensional perspective is more useful for capturing specific moral disengagement strategies and how they are employed in particular situations. Furthermore, a multi- dimensional perspective is consistent with Bandura's (2016) theoretical proposition that moral disengagement cannot be assessed unidimensionally. In this regard, Bandura (2016) states that:

Moral disengagement is not a dispositional trait that can be assessed by a one- size-fits-all measure. Disengagement mechanisms operate across different aspects of life, but they are manifested differently depending on the sphere of activity ENT#091;...ENT#093; Development of valid measures requires thorough understanding of how disengagement mechanisms are manifested in given spheres of activity (p. 26).

The above provides theoretical support for the psychometric evidence of the construct's multidimensionality and usefulness in measuring the socio-cognitive mechanisms of moral disengagement in justice-involved youth. Additionally, a reliable measure would be useful for SRPA programs in Colombia to assess changes in moral dis- engagement cognitions during reeducation processes and promote prosocial moral reasoning, emotional regulation, and empathy in young people who have engaged in delinquent behaviors (Agudelo Rico et al., 2024; Durán et al., 2025; Gómez Tabares et al., 2025a) . This is particularly important given the meta-analytic evidence of the relationship between moral disengagement and delinquent behavior in adolescents (Férriz Romeral et al., 2019).

This study has several limitations. Data were collected using self-report measures, which are susceptible to social desirability bias and possible distortion of responses, particularly among justice-involved youth in custodial settings. The correctional context is characterized by the regulation of morally transgressive attitudes and behaviors, which could reduce the likelihood of exhibiting moral disengagement cognitions. Future research should incorporate a social desirability scale as a control variable and include additional informants, such as educators and psychosocial teams from the institutions, to improve the validity of the results.

While this study included a multicenter sample of justice-involved youth from various specialized centers in Colombia, the results cannot be generalized to non-delinquent populations or groups with different sociocultural backgrounds. This limitation underscores the research gap that motivated the present study and highlights its main contribution-providing empirical evidence on the measurement of moral disengagement among justice-involved youth. Since moral disengagement is influenced by psychological and cultural factors, future studies with cross-cultural samples are necessary to confirm the factorial stability of the proposed models.

Despite these limitations, this study provides valuable information on the factorial structure and validity of the Moral Disengagement Scale (MMDS) for assessing the construct in justice-involved youth in Colombia and corroborates the role of moral disengagement in antisocial and delinquent behaviors.
